# Digital Versus Conventional Appliance Fabrication in Orthodontics: A Prospective Cohort Study on Students’ Perception of Education

**DOI:** 10.1002/jdd.70113

**Published:** 2025-12-04

**Authors:** Lisa‐Marie Mai, Jonas Q. Schmid, Anja Quast, Philipp Meyer‐Marcotty, Bernhard Wiechens

**Affiliations:** ^1^ Department of Orthodontics University Medical Center Göttingen Göttingen Germany; ^2^ Department of Orthodontics University of Münster Münster Germany

**Keywords:** dental, education, orthodontic, user experience

## Abstract

**Aim:**

This prospective cohort study aimed to evaluate the subjective perception of digital and conventional orthodontic fabrication methods among dental students. The educational effectiveness of conventional and digital approaches was assessed using a novel approach that included user experience surveys and a quantitative evaluation of the teaching units.

**Methods:**

A total of 64 pre‐graduated dental students from two consecutive semesters independently fabricated a conventionally and a digitally manufactured orthodontic appliance. Subjective evaluations were collected about the two different fabrication methods using standardized User Experience Questionnaires (UEQ). Six dimensions were assessed: attractiveness, perspicuity, efficiency, dependability, stimulation, and novelty. The corresponding teaching unit (CAD/CAM seminar) was evaluated using a Likert‐based questionnaire. The statistical analysis included Wilcoxon tests for paired samples and reliability assessments (Cronbach's alpha, Guttman's lambda‐2).

**Results:**

The digital workflow received higher ratings in all UEQ dimensions, particularly for novelty (*M* = 1.87 vs. −1.08, *p* < 0.0001). Attractiveness of digital methods was perceived positively compared to the conventional approach (*M* = 1.59 / *M* = −0.21). Pragmatic quality (perspicuity, efficiency, and dependability) was also rated more favorably for the digital approach (*M* = 0.88 / 0.12). The associated CAD/CAM seminar received an overall positive evaluation. Participants reported learning progress and expressed interest in continuing the course.

**Conclusion:**

Digital orthodontic fabrication methods are perceived more positively than conventional techniques by dental students, highlighting the value of integrating digital workflows into dental education.

## 1 Introduction

Digital technologies, such as CAD/CAM (Computer‐aided design/manufacturing), intraoral scanning, and 3D imaging are shaping orthodontic practice and training [1, 2, 3]. Compared to conventional methods, digital workflows offer advantages in terms of accuracy, efficiency, and patient communication. Aligners, indirect bonding trays, and splints are swiftly produced in‐office [4, 5].

This digital transformation also impacts dental education. Students must acquire both conventional manual skills (e.g., fabricating functional appliances) and digital competencies to meet clinical demands. Curricula are increasingly integrating both components, but how students subjectively perceive these technologies remains to be studied.

While clinical competence remains the primary goal of orthodontic education, evaluating students’ learning experiences offers valuable insight into how educational methods can be optimized. As digital workflows are introduced not as replacements but as complementary teaching components, understanding these perceptions is particularly relevant.

Educational research has shown that digital manufacturing tools, like 3D‐printed dental models and virtual aligner setups, can improve spatial understanding, motivate learning, and increase awareness of innovation [6, 7]. Institutions have responded by offering structured 3D simulation modules and digital seminars to promote clinical readiness [8]. Dental students generally rate digital techniques as more intuitive, less stressful, and easier to use than conventional workflows [9, 10, 11, 12, 13]. Clinically, digital procedures have also demonstrated comparable accuracy and reduced chairside time [14]. Nevertheless, limited access, steep learning curves, and high costs remain challenging [15, 16].

Beyond the techniques themselves, teaching formats and assessments can influence learning outcomes. Randomized trials indicate that structured grading may improve appliance quality compared to pass/fail formats [17]. Furthermore, hybrid learning approaches combining digital and in‐person teaching are valued for their flexibility and effectiveness [18, 19]. Studies on digital teaching formats report increased comprehensibility and engagement, especially when digital technologies are effectively embedded in clinical education [20, 21].

Overall, dental students welcome digital learning. However, studies indicate that aspects including teacher training, greater interactivity, and alignment of lecture length to students' attention spans could be improved [22].

Altogether, these results indicate a necessity to evaluate not only the technical performance but also the subjective user experience of digital workflows in dental education. For this purpose, the User Experience Questionnaire (UEQ) [23] was used in the present study to compare students’ subjective evaluations of digital versus conventional orthodontic workflows. The associated seminar (CAD/CAM seminar) was also evaluated in terms of teaching quality. The aim of this study was to evaluate the user experience in relation to both manufacturing methods and the teaching format. The null hypothesis (H_0_) stated that there is no significant difference between digital and conventional workflows in any UEQ dimension. It should be noted that the present study compares two distinct educational workflows: conventional fabrication of a functional activator and the digital workflow of a centric splint. These procedures differ in their clinical objectives and complexity, which may influence students’ subjective evaluations. The focus of the study is therefore primarily educational rather than clinical in nature.

## 2 Material and Methods

This study was reviewed and approved by the appropriate institutional review board (IRB). In this prospective cohort study, 64 dental students from two consecutive semesters were introduced to both manufacturing methods. Each participant independently fabricated a conventional functional orthodontic appliance (activator) and a digitally produced centric splint as part of a CAD/CAM seminar. The students fabricated the orthodontic appliances during the scheduled teaching period of each semester. The fabrication period spanned from April to July 2024 in the summer term, and from October 2024 to February 2025 in the winter term, commencing with the start of the respective CAD/CAM course. Afterwards, they evaluated both fabrication processes using a standardized questionnaire based on the UEQ.

### 2.1 Study Design

The objective was to assess the subjective evaluation of two manufacturing processes (conventional vs. digital) using standardized questionnaires. Data collection was anonymous and paper‐based at the end of each semester. In addition to assessing both workflows, students also evaluated the CAD/CAM seminar in terms of clarity, organization, and perceived learning success.

**FIGURE 1:**
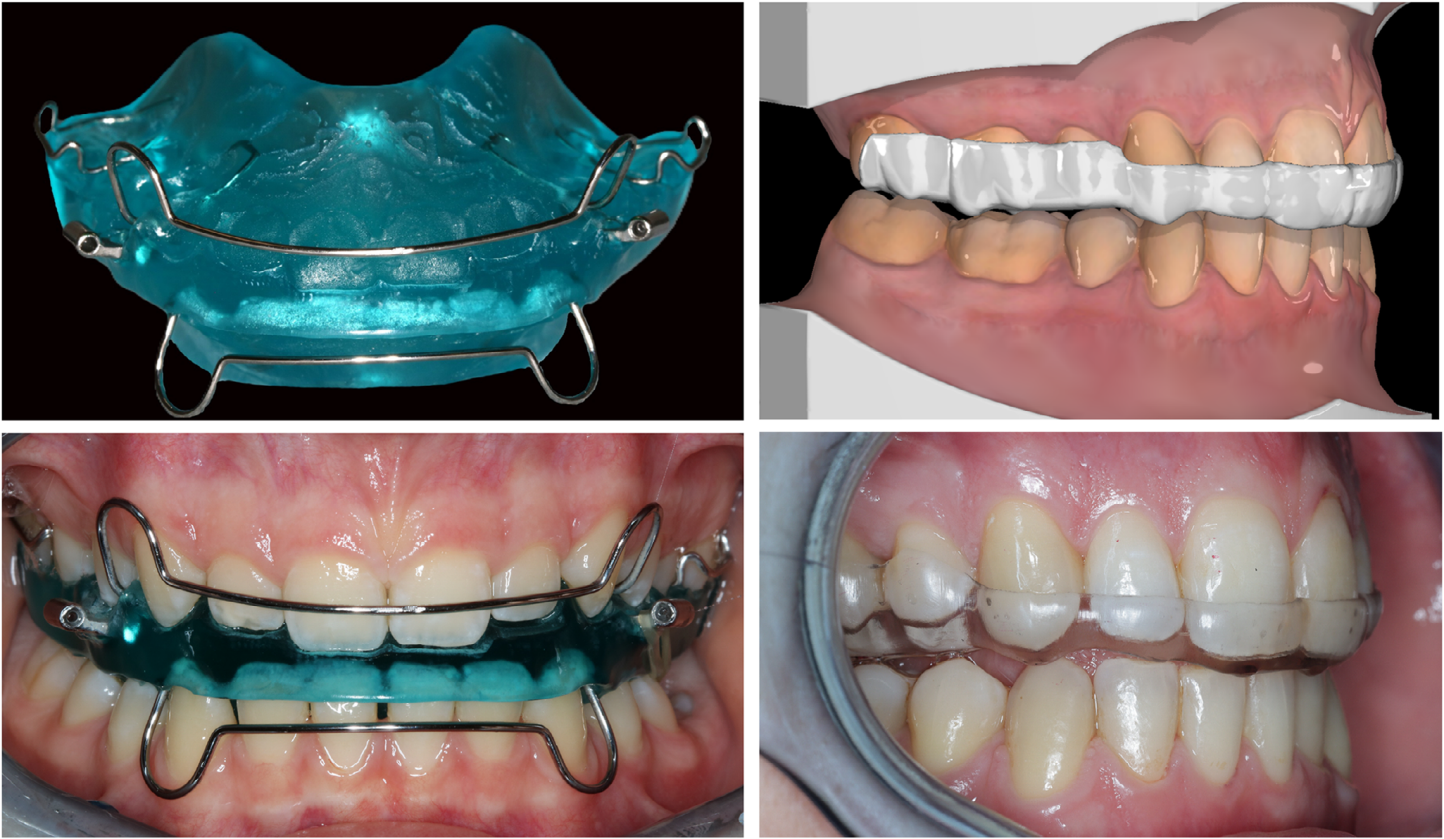
Conventional fabrication—PMMA addition process (left): Orthodontic functional appliance (activator); digital workflow (right): CAD/CAM manufactured centric splint.

The students fabricated a conventional functional orthodontic appliance (activator) using model casts and a standardized monomer‐based technique, including the PMMA addition process. As a parallel procedure, a digitally supported workflow was used to plan and produce a centric splint based on intraoral scan data (Trios 4, 3Shape, Denmark) and 3D modeling software (OnyxCeph, Image Instruments GmbH, Germany) (Figure 1). The splints were printed using a 3D printer (Form 3B+, Formlabs Inc., USA). Both phases were accompanied by theoretical instruction and supervised practical training.

### 2.2 Participants and Inclusion Criteria

A total of 64 dental students participated in the study. Since each student completed separate evaluations for both workflows, the dataset included 109 valid questionnaires (digital: *N* = 56, conventional: *N* = 53), while questionnaires with incomplete or invalid responses were excluded.

To be included in the study, the students had to be enrolled in the respective semester (summer term 2024 and winter term 2024/25), attend the CAD/CAM seminar regularly, and independently fabricate both the conventional and the digital orthodontic appliance. In addition, they were required to complete the evaluation questionnaires at the end of the semester. Students who did not meet these criteria were excluded from the analysis. The sample size was determined by the number of dental students enrolled in the respective semesters and participating in the CAD/CAM seminar during the study period.

Gender and age data were not provided by all participants. For the digital workflow, 42 students reported gender (29 female), and 41 reported age (24.71 years, min = 22, max = 33). For the conventional workflow, 43 students reported gender (27 female) and 42 reported age (24.93 years, min = 22, max = 33). An additional evaluation of the CAD/CAM seminar itself included a sample of 50 students (33 female) (Table 1).

**TABLE 1:** Baseline characteristics of study participants.

Group	Mean age (range)	Female (*N*)	Male (*N*)
Digital	24.71 (22–33)	29	13
Conventional	24.93 (22–33)	27	16

### 2.3 Outcome Measures

The primary outcome of this study was the subjective evaluation of digital versus conventional workflows by dental students. This was assessed using the six scales of the German version of the UEQ. The H_0_ assumed no significant differences between the two manufacturing methods across these dimensions.

The secondary outcome focused on the evaluation of the CAD/CAM seminar itself. This evaluation aimed to assess the overall educational effectiveness of the seminar.

### 2.4 Survey Instrument

A standardized questionnaire based on the UEQ was used to assess students’ subjective evaluations [23]. The UEQ is a validated instrument widely used in both research and industry to assess various dimensions of user experience, including usability. It uses bipolar adjective pairs rated on a 7‐point Likert scale. The following six scales were included in this study:

•Attractiveness (e.g., unpleasant–pleasant)
•Perspicuity (e.g., confusing–clear)
•Efficiency (e.g., inefficient–efficient)
•Dependability (e.g., unpredictable–predictable)
•Stimulation (e.g., boring–exciting)
•Novelty (e.g., conservative–innovative)

The scale values range from −3 (*extremely negative*) to +3 (*extremely positive*). Values between −0.8 and +0.8 are interpreted as neutral, values above +0.8 as positive, and values below −0.8 as negative.

### 2.5 Evaluation of the CAD/CAM Seminar

In addition to comparing the two manufacturing processes, the CAD/CAM seminar itself was evaluated in terms of didactic and practical quality. Students rated, among other things, the clarity of instruction, their perceived learning gain, and their overall satisfaction with the organization and execution of the course. These aspects were assessed using a 5‐point Likert scale (1 = *strongly agree*, 5 = *strongly disagree*) and were analyzed descriptively.

### 2.6 Data Analysis

Internal consistency was tested using Cronbach's alpha and Guttman's lambda‐2. After checking for normal distribution (Shapiro–Wilk test), the comparisons between both manufacturing methods were evaluated using Wilcoxon matched‐pairs signed rank tests. The significance level was set at *p* < 0.05. Statistical analysis was performed using GraphPad Prism, Version 10.4.2 (534) (GraphPad Software, LLC, San Diego, California, USA).

Given the within‐subjects design, each participant rated both workflows, resulting in paired observations. The Wilcoxon matched‐pairs signed‐rank test was therefore chosen as the appropriate non‐parametric alternative to the paired‐samples *t*‐test, since several UEQ scale variables deviated from normal distribution.

In addition, a post‐hoc correlation analysis (Spearman's *ρ*) was performed to examine potential associations between participants’ age and each of the six UEQ scale scores for both workflows. This analysis aimed to evaluate whether age differences could have influenced subjective user experience ratings.

Only students who had completed both questionnaires (conventional and digital) were included in the statistical analysis. After excluding cases with only one valid response, the final dataset comprised 53 students, each providing one conventional and one digital evaluation.

## 3 Results

For all six UEQ scales of both the digital and conventional workflows, internal consistency showed reliability ranging from questionable to excellent. Cronbach's alpha ranged from 0.71 to 0.90 for the digital workflow, indicating acceptable to excellent reliability, and from 0.65 to 0.90 for the conventional workflow, indicating questionable to excellent reliability. Guttman's lambda‐2 (*λ*_2_) ranged from 0.72 to 0.90 (digital), indicating acceptable to excellent reliability, and from 0.68 to 0.89 (conventional), indicating questionable to good reliability.

According to the Shapiro–Wilk test, 8 out of the 12 variables tested met the assumption of normal distribution, while four deviated significantly (*p* < 0.05). To assess significant differences between the two groups, a Wilcoxon matched‐pairs signed rank test was conducted for each UEQ scale. A statistically significant difference was found for the scale Novelty (*p* < 0.0001). For all other scales (Perspicuity, Efficiency, Dependability, Stimulation, Attractiveness), no significant differences were observed (all *p* > 0.05) (Table 2).

**TABLE 2:** Descriptive statistics (M, SD) and Wilcoxon matched‐pairs signed rank test results for UEQ scales.

UEQ scale	Digital M (SD)	Conventional M (SD)	Wilcoxon W	*p* value
Perspicuity	4.08 (0.44)	3.96 (0.47)	−60.0	0.7005
Stimulation	4.11 (0.38)	3.96 (0.48)	−245.0	0.0852
Dependability	4.05 (0.49)	3.88 (0.60)	−247.0	0.0720
Attractiveness	3.80 (0.36)	3.91 (0.39)	149.0	0.3384
Efficiency	4.25 (0.55)	4.34 (0.73)	188.0	0.2252
Novelty	4.23 (0.51)	3.61 (0.57)	−803.0	**< 0.0001**

*Note*: Mean values (M) and standard deviation (SD) are reported for each User Experience Questionnaire (UEQ) scale, comparing the digital and conventional workflow. The values represent the raw means on the original 1–7 Likert scale used in the UEQ for descriptive transparency. In the main text and figures, the corresponding standardized UEQ scores (−3 to +3) are presented according to the official UEQ evaluation framework. The Wilcoxon matched‐pairs signed rank test was used to evaluate differences between the two groups. Bold values indicate statistically significant differences (*p* < 0.05).

### 3.1 Comparison of Digital and Conventional Workflows

**FIGURE 2:**
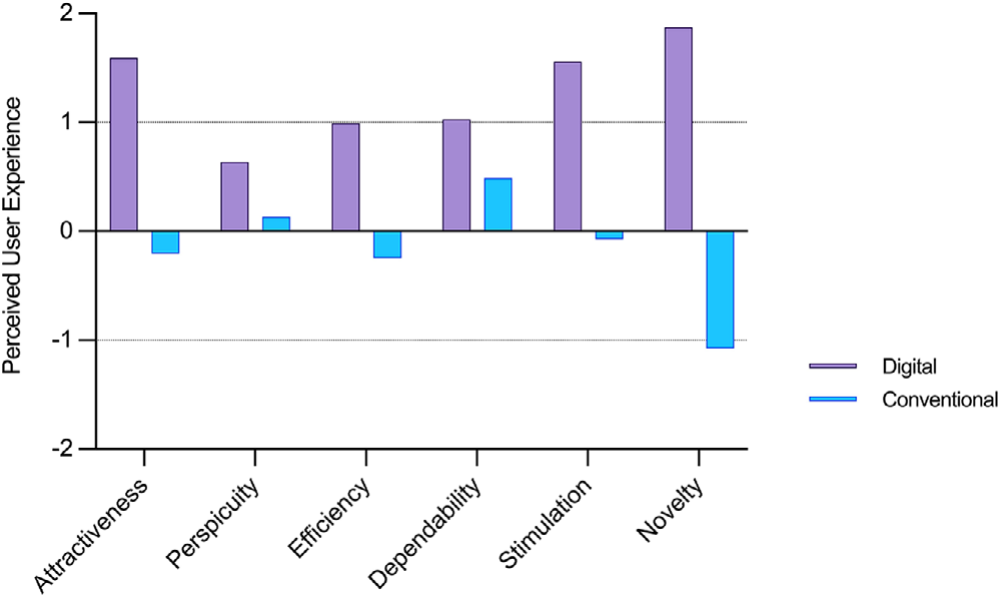
Perceived user experience across UEQ scales for the digital and conventional group.

**FIGURE 3:**
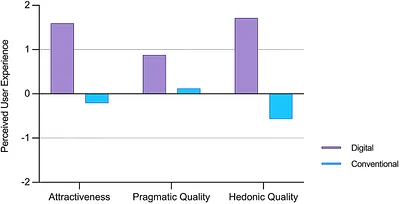
Perceived user experience across UEQ dimensions for the digital and conventional group.

**FIGURE 4:**
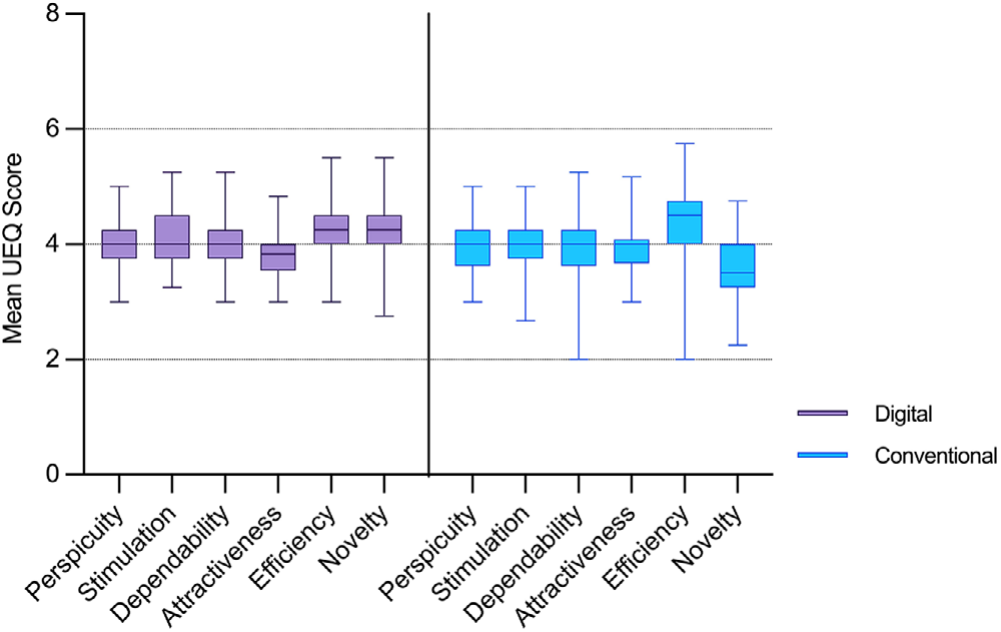
Boxplot diagrams showing the mean, minimum, and maximum values for each scale of the User Experience Questionnaire (UEQ), comparing the digital and conventional workflow in orthodontic appliance fabrication.

*Digital Workflow*: The Attractiveness scale showed a clearly positive perception (*M* = 1.59, Var = 1.14). Pragmatic quality, composed of the scales Perspicuity, Efficiency, and Dependability, had an overall mean of *M* = 0.88. Among these, Dependability received the highest rating (*M* = 1.03, Var = 0.96), followed by Efficiency (*M* = 0.99, Var = 1.48) and Perspicuity (*M* = 0.63, Var = 1.62). Hedonic quality, which consists of Stimulation and Novelty, was also rated highly (*M* = 1.71). Within this dimension, both Stimulation (*M* = 1.56, Var = 1.43) and Novelty (*M* = 1.87, Var = 0.90) received particularly favorable evaluations (Figures 2, 3, 4).

*Conventional Workflow*: The Attractiveness scale was rated as rather neutral to slightly negative (*M* = −0.21, Var = 1.22). With regard to pragmatic quality, the overall mean was *M* = 0.12. Among these, Dependability achieved the highest rating (*M* = 0.49, Var = 1.19), whereas Perspicuity received a moderate score (*M* = 0.13, Var = 1.21) and Efficiency was evaluated negatively (*M* = −0.25, Var = 0.99). Hedonic quality was rated lower overall (*M* = −0.57), with Stimulation remaining close to a neutral perception (*M* = −0.07, Var = 1.77) and Novelty being assessed most critically (*M* = −1.08, Var = 1.33) (Figures 2, 3, 4).

Post‐hoc correlation analysis between age and UEQ scales showed no significant associations (all *p* > 0.05) for both the digital and conventional workflow (Table 3).

**TABLE 3:** Spearman correlations between age and UEQ scales for digital and conventional workflows.

	Digital		Conventional	
UEQ scale	*ρ*	*p* value	*ρ*	*p* value
Perspicuity	−0.11	0.52 (n.s.)	0.23	0.17 (n.s.)
Stimulation	−0.19	0.27 (n.s.)	0.20	0.23 (n.s.)
Dependability	−0.05	0.76 (n.s.)	0.13	0.43 (n.s.)
Attractiveness	0.11	0.52 (n.s.)	0.13	0.45 (n.s.)
Efficiency	−0.14	0.41 (n.s.)	−0.08	0.63 (n.s.)
Novelty	−0.00	> 0.99 (n.s.)	0.21	0.22 (n.s.)

*Note*: Spearman analysis results of the correlation between age and all six UEQ scales. Spearman's rho (*ρ*) and corresponding *p *values are reported.

Abbreviation: n.s., not significant.

### 3.2 Evaluation of the CAD/CAM Seminar

**FIGURE 5:**
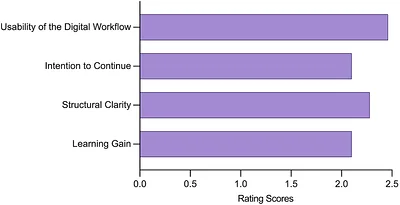
Average rating scores from the evaluation of the CAD/CAM seminar.

The evaluation of the CAD/CAM seminar showed the following mean scores: perceived learning gain was rated at 2.1 points, the structure of the seminar received a mean score of 2.28 points, and the interest in continuation was rated at 2.1 points. The ability to describe a digital workflow was rated at 2.46 points (Figure 5).

## 4 Discussion

The results of this study indicate that students rated the digital fabrication of orthodontic appliances more positively than the conventional method, particularly in the hedonic dimensions of Stimulation and Novelty (*M* = 1.71 vs. −0.57). This aligns with previous research emphasizing the engaging and user‐friendly nature of digital technologies in dental education [1, 2, 6, 10, 11].

In terms of pragmatic quality (Perspicuity, Efficiency, and Dependability), digital workflows were also rated higher overall (*M* = 0.88 vs. 0.12), likely reflecting students’ increasing familiarity with digital tools [24]. The Attractiveness scale, summarizing general user experience [23], similarly favored digital fabrication (*M* = 1.59 vs. −0.21), supporting findings that clarity and aesthetics influence acceptance [4].

Among all UEQ dimensions, only Novelty showed a statistically significant difference, indicating that the digital workflow was perceived as particularly innovative. The absence of significance in other areas suggests a generally comparable perception of core usability features, potentially shaped by individual preferences or familiarity with each technique.

In addition, the subjective results reflect broader trends seen in recent research on digital teaching formats, which reported that while students considered e‐learning tools useful and effective, they indicated a need for improved usability, learnability, and overall satisfaction [21].

An additional advantage of digital workflows is their inherent compatibility with remote or hybrid learning. Compared to conventional workflows, digital methods, such as virtual design or 3D planning, can be more easily integrated into online teaching modules. This flexibility aligns well with findings that hybrid learning formats are particularly valued for their adaptability and effectiveness [18, 19]. Furthermore, embedding digital components meaningfully into clinical education improves students’ clarity of understanding and engagement [20, 21], suggesting that digital orthodontic training offers not only technical and perceptual benefits but also broader didactic potential for future hybrid curriculum models.

Regarding the evaluation of the CAD/CAM seminar, the students reported positive learning gains and expressed interest in continuation. However, suggestions for improved content depth and structure point to the need for more hands‐on activities and better alignment with prior knowledge.

### 4.1 Strengths and Limitations

A key strength of this study is the within‐subject comparison of digital and conventional workflows under real teaching conditions, allowing for a direct and practical evaluation. The use of a validated tool (UEQ) enabled a structured assessment of user experience across multiple dimensions. Including the CAD/CAM seminar evaluation added educational context and highlighted students’ expectations.

However, several limitations should be discussed. First, the two types of appliances compared are varied, which limits clinical comparability but reflects the different educational workflows encountered in modern dental curricula by contrasting students’ experiences with conventional and digital fabrication methods. Second, the conventional activator fabrication involved more complex manual steps and material handling than the digital splint workflow, potentially influencing students’ perceptions of efficiency and workload. Third, the relatively wide age range of participants may have influenced their familiarity with digital tools and, consequently, their subjective user experience. However, the post‐hoc correlation analysis revealed no significant relationship between age and any UEQ dimension, suggesting that age‐related effects were minimal in this cohort.

Finally, the study is also limited by its sample size and restriction to two semesters at a single institution. Perceptions were assessed after one‐time exposure, without long‐term follow‐up or objective performance data. Uncontrolled factors, such as prior digital skills or supervisor influence, may have affected the results. Future research should combine subjective experience with objective outcomes and track changes over time.

### 4.2 Implication and Future Directions

User experience plays a vital role in adopting digital tools in dental education. While positive perceptions alone do not ensure long‐term use, they are critical for student engagement and willingness to adopt new techniques. The findings support a more widespread integration of digital workflows, including their potential in hybrid and online educational formats.

Curricula should aim to balance manual and digital skill development and use both formats to reinforce learning. As technologies advance and students’ expectations evolve, dental education must remain adaptive, supported by structured evaluation tools like the UEQ and by high‐quality, well‐designed digital learning environments.

## 5 Conclusion

The results of this study demonstrate that digital workflows in orthodontic education are perceived more positively by students than conventional techniques, especially in terms of a higher hedonic quality.

These findings suggest that digital technologies can not only improve processes but also enhance motivation and user experience. Pragmatic qualities were also rated higher for digital methods, though conventional techniques were sometimes valued for greater controllability, which implies that digital workflows are beneficial from both a technical and educational standpoint. Rather than replacing traditional methods, they should be integrated alongside them as complementary components of modern dental curricula.

## Funding

The authors have nothing to report.

## Conflicts of Interest

The authors declare no conflicts of interest.
